# COVID‐19‐associated pityriasis rosea in children: Case report and literature review

**DOI:** 10.1002/ccr3.6096

**Published:** 2022-07-19

**Authors:** Maryam Khalili, Bahareh Abtahi‐Naeini, Fereshte Rastegarnasab, Kimia Afshar

**Affiliations:** ^1^ Pediatric Dermatology Division of Department of Dermatology, Afzalipour Hospital Kerman University of Medical Sciences Kerman Iran; ^2^ Pediatric Dermatology Division of Department of Pediatrics, Imam Hossein Children's Hospital Isfahan University of Medical Sciences Isfahan Iran; ^3^ Skin Diseases and Leishmaniasis Research Center Isfahan University of Medical Sciences Isfahan Iran; ^4^ Student Research Committee Isfahan University of Medical Sciences Isfahan Iran

**Keywords:** child, COVID‐19, cutaneous, pityriasis rosea, SARS‐COV‐2, skin

## Abstract

Skin lesions are one of the Coronavirus disease 2019 (COVID‐19) symptoms. Pityriasis rosea (PR) is a mucocutaneous manifestation that can occur following virus infections. Most of the PR lesions after COVID‐19 infection were reported in adults. Herein, we report a child with PR lesions, and a literature review on 5 other case reports in children.

## 1 INTRODUCTION

The Coronavirus disease 2019 (COVID‐19) symptoms are variable; but often include fever, cough, and fatigue.^1^ Severe acute respiratory syndrome Coronavirus 2 (SARS‐CoV‐2) infection can also present with cutaneous manifestations classified into two major categories: inflammatory and vasculopathy lesions.

The clinical presentation, course, outcome, and cutaneous manifestations of SARS‐CoV‐2 infection in children usually differ from adults.^2^

One uncommon COVID‐19‐related mucocutaneous manifestation is pityriasis rosea (PR)^2^; typically presenting with a single, erythematous plaque followed by a secondary eruption with lesions on the cleavage lines of the trunk (configuration of a “Christmas tree”).^3^

As we reviewed, most of the reported cases of PR lesions after COVID infection are adults^4^, ^5^ and PR is quite rare in children under 10 years old.^6^ Herein, we report a case of PR during the post‐COVID period in a 7‐year‐old child.

## 2 CASE PRESENTATION

**FIGURE 1:**
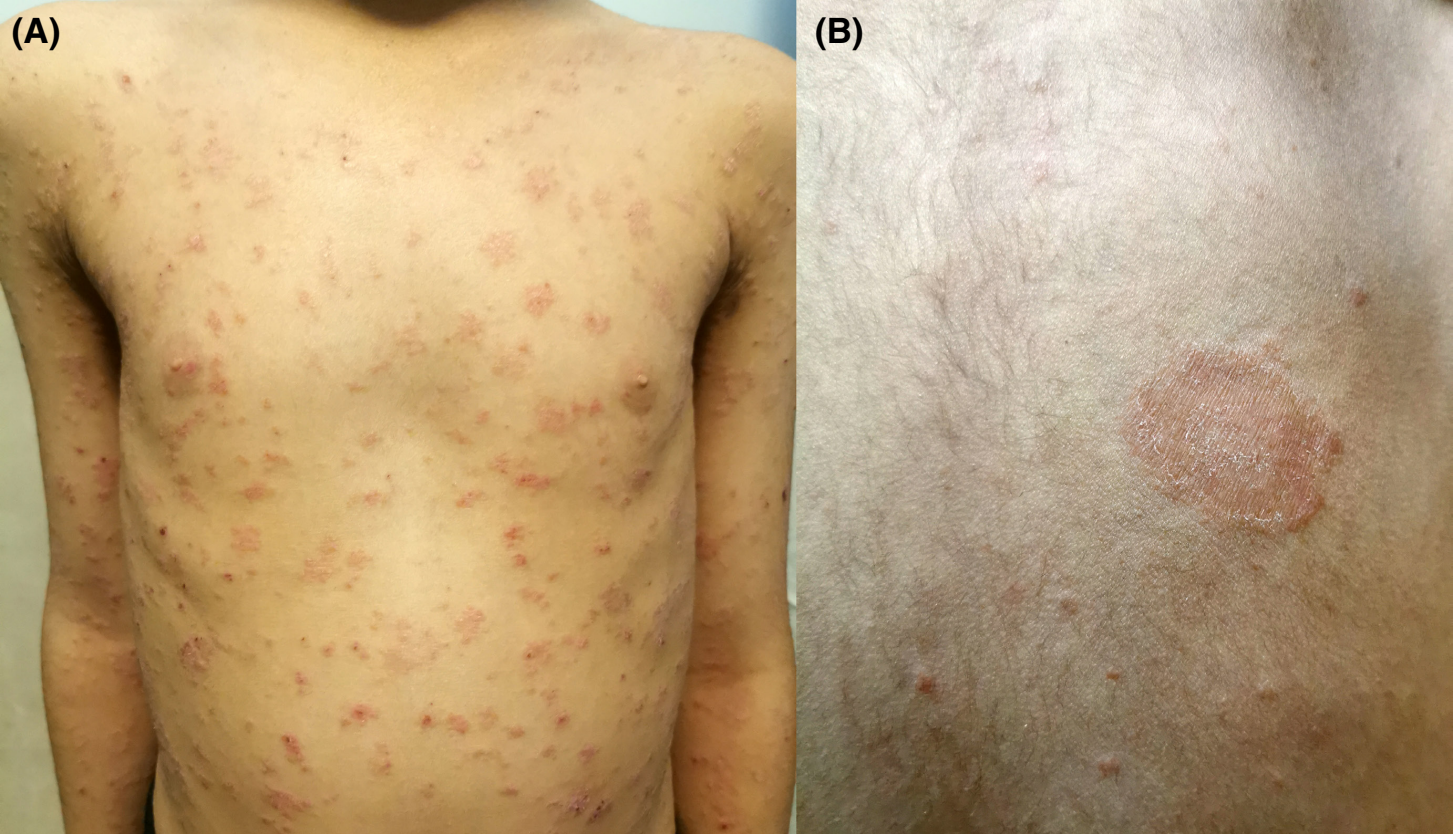
COVID‐19 associated Pityriasis rosea‐like eruptions. Several erythematosquamous patches and plaques are located on the trunk of a child (A). Larger, slightly raised, scaly plaque on the back (B).

A 7‐year‐old girl presented with erythematous scaly patches on the anterior and posterior of the trunk, in association with a larger herald patch on the back (Figure 1). The patient had no symptoms other than itching at this period. Two weeks before the initiation of cutaneous lesions, she suffered from cough, sore throat, rhinorrhea, and mild diarrhea. She had no remarkable past medical history. At the same time, the patient and her family had the same symptoms with positive RT‐PCR for SARS‐CoV‐2 infection. Based on her clinical presentations and history of confirmed COVID‐19, a diagnosis of COVID‐19‐associated Pityriasis rosea was made. Topical corticosteroids and systemic antihistamines were started for treating pruritic patches. Clinical improvement appeared 2 weeks after the initiation of conservative treatment.

## 3 DISCUSSION

We reported a 7‐year‐old girl with SARS‐CoV‐2‐related PR during the post‐infection period.

The exact etiopathogenesis of PR is still unknown; but in many cases, before the onset of PR, there are episodes of upper respiratory tract infections, that highlight the viral etiology of this condition.^7^

Viruses, vaccination, and drugs have been implicated as the cause of PR. The most important viruses are Human Herpesvirus (HHV)‐6 and HHV‐7. These viruses may also interact with each other, explaining recurrences and atypical presentations.^8^

Pityriasis rosea and PR‐like eruptions have been reported following other viruses vaccines like influenza, poliomyelitis, yellow fever, hepatitis A, rabies, and Japanese encephalitis.^9^

It seems that COVID‐19 can be a trigger for PR. In COVID‐19 cases, the skin lesions appear due to a large invasion of pro‐inflammatory cytokines and micro‐thrombosis on the skin.^6^

Overall, SARS‐CoV‐2 may have triggered a chain viral reaction. SARS‐CoV‐2 may have played a trans‐activating role; triggering HHV‐6, HHV‐7, and Epstein–Barr virus (EBV) reactivation and causing cutaneous PR‐like lesions. EBV concurrent systemic reactivations have been detected in a patient with PR and COVID‐19.^10^

Also, the psychological stress linked to the pandemic and the immunosuppression associated with SARS‐CoV‐2 infection may enable the reactivation of latent viral infections.^8^

Dermatologic symptoms of COVID, like other symptoms, are less severe in children than in adults.^11^ PR lesions in adults can present as erythematous plaques or few patches disseminating after a few days to a wider surface of the body.^5^, ^6^, ^7^ Also these lesions in reported adults mostly last more than 2 weeks^4^, ^5^, ^6^, ^12^; which is a long time compared with reported children.^11^, ^13^, ^14^ The pruritic lesions were treated conservatively with an antihistamine or topical corticosteroids in adults^4^, ^5^, ^6^, ^7^, ^12^ and children.^10^, ^11^

To date, five cases of PR and PR‐like eruption related to COVID‐19 in children have been published (Table 1).^10^, ^11^, ^13^, ^14^, ^15^ In these cases, the patient’s ages were between 5 and 16 years old; three of them had mild symptoms of COVID‐19 and had close contact with confirmed cases.^10^, ^11^, ^15^ All of them had erythematous scaly patches on the trunk and extremities, which lasted for 10–14 days. They had conservative treatment; but only in one case, systemic steroid therapy was considered.^13^

**TABLE 1:** Reported cases of COVID‐19 associated pityriasis rosea (PR) in children

No.	Author/Year	Age/Sex	Manifestations of COVID‐19	Dermatologic manifestation	Duration of cutaneous lesions	PCR	Exposure to COVID‐19 patients	Treatment for PR
1	Francesco Drago/2020^10^	16 year/Male	Fever, headache, fatigue, arthralgias, myalgias, loss of appetite	Oval erythematous papulosquamous lesions in the typical “Christmas tree” observed over the trunk, eruption preceded by a single scaly oval patch on the abdomen	4 weeks	P	Three weeks earlier, the mother and father of the patient had COVID‐19	Conservative
2	Sze May Ng/2020^14^	12 year/Male	Fever, sore throat, abdominal pain, diarrhea	Generalized maculopapular rash, herald patch noted on the back of the torso	2 weeks	P	NA	NA
3	Antonio Urbano Monteiro Neto/2020^11^	5 year/Male	NA	Sparse small plaques with an oval shape and little desquamation on the trunk	15 days	NA	Housekeeper had COVID‐19 15 days ago. His father diagnosed with coronavirus	Conservative
4	Maria Dakoutrou/2021^15^	7 year/Male	Mild abdominal pain, diarrhea	Erythematous scaly patches on the trunk and upper extremities and a typical herald patch on the right upper arm	NA	NA	Close contact with two COVID‐19 cases in the family	NA
5	Fabrizio Martora/2021^13^	16 year/Female	NA	Erythematous‐squamous papules and plaques with pruritus placed on the trunk	2 weeks	N	NA	Topical steroid and systemic antihistamine therapy without any result/Systemic steroid therapy
6	Our case	7 year /Female	Gastroenteritis, cough, sore throat, rhinorrhea	Erythematous scaly itchy patches on the trunk and a larger herald patch on the back	8 days	NA	The family had the same symptoms with positive PCR for COVID‐19	Topical steroid therapy and systemic antihistamine

Abbreviations: N, negative; NA, not available; P, positive.

## 4 CONCLUSION

COVID‐19 can cause pityriasis rosea as other systemic and cutaneous symptoms. Due to the mild symptoms of COVID‐19 in children, considering these cutaneous manifestations can guide to better diagnosis and care.

## AUTHOR CONTRIBUTIONS

B.A.‐N. provided the case. M.K. and B.A.N. contributed to designing and conducting the study. M.K. and B.A.N. contributed to the revised manuscript critically for important intellectual content. K.A. and F.R. assisted in the interpretation of data and the preparation of the first draft of the manuscript. All authors have read the final version and approved the content of the manuscript to be published and confirmed the accuracy or integrity of any parts of the work.

## CONFLICT OF INTEREST

The authors declare that there is no conflict of interest regarding the publication of this paper.

## CONSENT

Written informed consent was obtained from the patient to publish this report in accordance with the journal's patient consent policy.

## Data Availability

The data that support the findings of this study are not publicly available due to containing information that could compromise the privacy of our research participant but are available from our first author as requested.
